# An Unlikely Cause of Abdominal Pain

**DOI:** 10.5811/cpcem.2018.2.37073

**Published:** 2018-03-14

**Authors:** Norah Kairys, Keegan Skidmore, Jennifer Repanshek, Wayne Satz

**Affiliations:** Temple University Hospital, Department of Emergency Medicine, Philadelphia, Pennsylvania

## Abstract

Cecal bascule is a rare subtype of cecal volvulus where the cecum folds anterior to the ascending colon causing intestinal obstruction. It is a challenging diagnosis to make in the emergency department, as the mobile nature of the cecum leads to a great deal of variation in its clinical presentation. Our discussion of a 78-year-old female who presented with abdominal pain and was found to have a cecal bascule requiring right hemicolectomy, demonstrates how emergency physicians must expand their differential diagnosis for patients reporting signs of intestinal obstruction. Though cecal bascule does not present often, the need for early surgical intervention necessitates a high level of clinical suspicion to prevent life-threatening complications.

## INTRODUCTION

Most cases of large bowel obstruction worldwide are caused by malignancy, with diverticular disease and volvulus causing the small remainder of obstructions.^1^ Of the cases of intestinal volvulus, the sigmoid is the most commonly affected portion of the colon. 2 Cecal volvulus accounts for less than 1% of all large bowel obstructions, and a cecal bascule is estimated to occur in only 10% of these cases of cecal volvulus. 1

A review of 561 patients with cecal volvulus demonstrated that this condition commonly presents with symptoms of bowel obstruction – abdominal pain, constipation, nausea and vomiting.^3^ Physical exam may reveal abdominal distension, hyperperistalsis, peritoneal signs, an abdominal mass and/or absent bowel sounds.^3^ However, patients with a cecal bascule may only have intermittent signs of obstruction due to periodic flipping of the cecum back into its anatomical position.^1^ Many cases of cecal bascule presenting to the emergency department (ED) are missed on initial presentation due to this phenomenon of periodic obstruction. This subtype is thought to strangulate less often as its mesentery is infrequently twisted, but it can still progress to cause intestinal ischemia if not adequately addressed.^1^

## CASE REPORT

A 78-year-old female with recent placement of a percutaneous endoscopic gastrostomy (PEG) presented to the ED with a chief complaint of abdominal pain over the prior two days. The patient stated that the pain had begun at her PEG-tube site after putting pressure on the area when climbing out of a car the day before. The pain was intermittent and cramping in nature – lasting a few minutes before subsiding and becoming more severe over the previous 24 hours. At the time of presentation, the patient was complaining of some abdominal distension but denied any nausea, vomiting, constipation, or obstipation.

On exam, her vital signs were mildly abnormal, but did not demonstrate a patient who was hemodynamically unstable. Her respiratory rate was slightly elevated at 20 breaths per minute, her heart rate was 97 beats per minute, she had a pulse oximeter reading of 94% on room air, and she was afebrile. The patient’s blood pressure was also within the normal range at 115/85 millimeters of mercury. On physical examination, the patient’s abdomen was soft but distended, and diffusely tender to palpation.

**Image 1:**
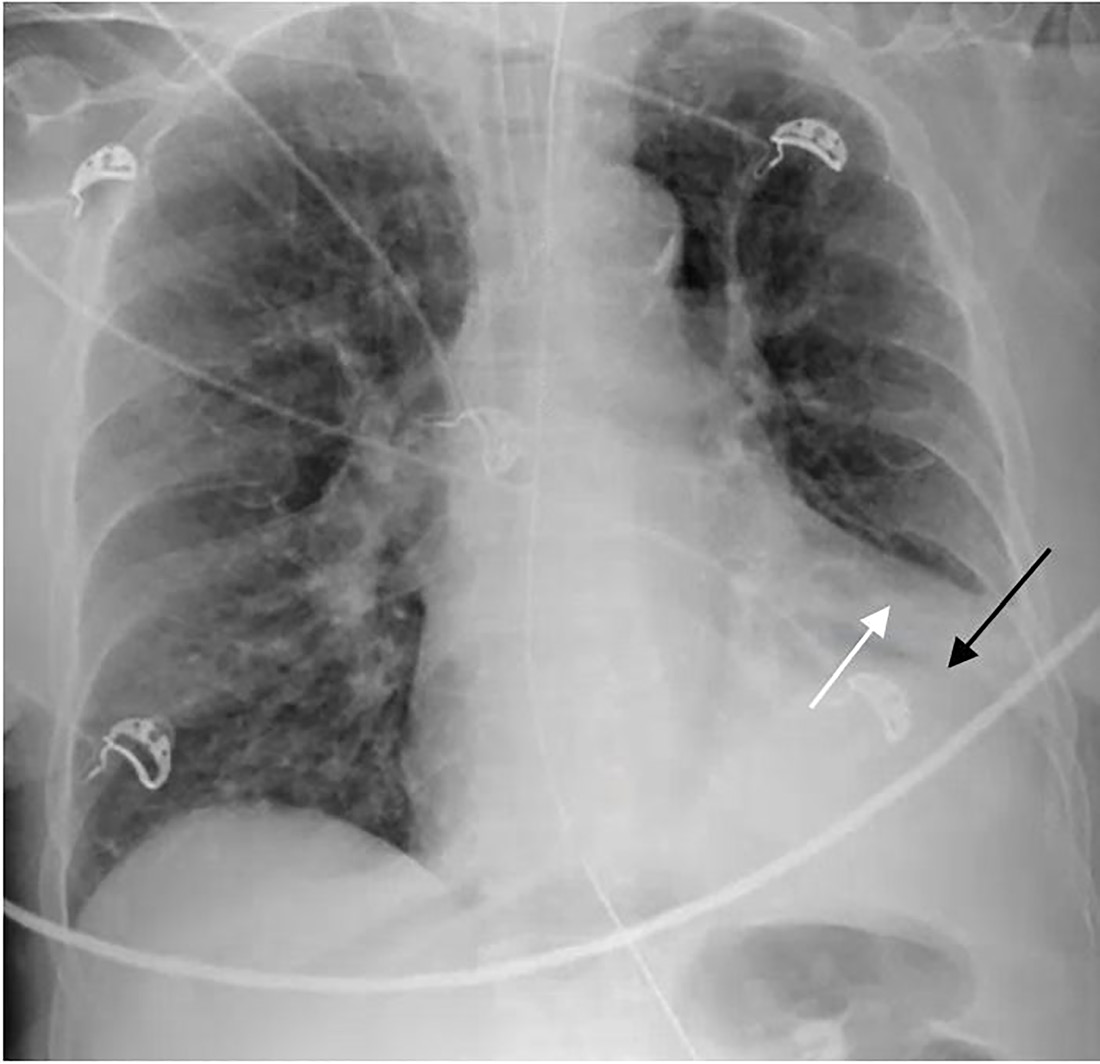
Posterior-anterior radiograph of the chest demonstrating elevation of left hemi-diaphragm (black arrow) and left lower lobe consolidation (white arrow).

While awaiting the results of her bloodwork, she had one episode of non-bloody bilious emesis in the ED. She was found to have a serum potassium level of 2.9 mEq/L, chloride of 95 mEq/L, bicarbonate of 36 mEq/L, and a pH of 7.49. Interestingly, her white blood cell count was 6.2 * 10^9^/L. Her chest radiography demonstrated elevation of the left hemi-diaphragm and left lower lobe consolidation (Image 1).

**Image 2:**
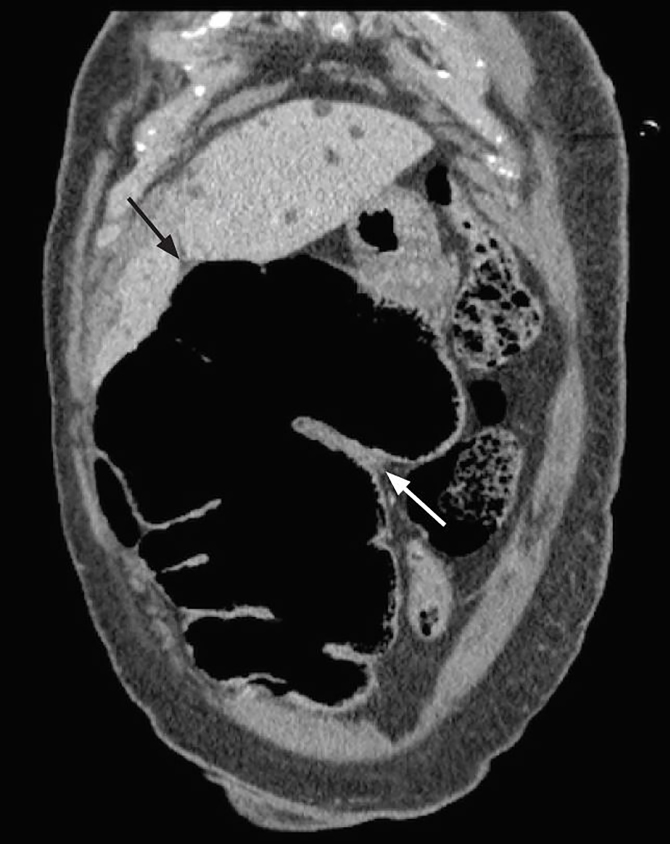
Computed tomography coronal image demonstrating marked gaseous distension of the distal cecum, which is flipped cephalad extending into the mid to right upper quadrant (black arrow). Comma sign (white arrow) refers to comma-shaped thickening of the root of the sigmoid mesocolon.

**Image 3:**
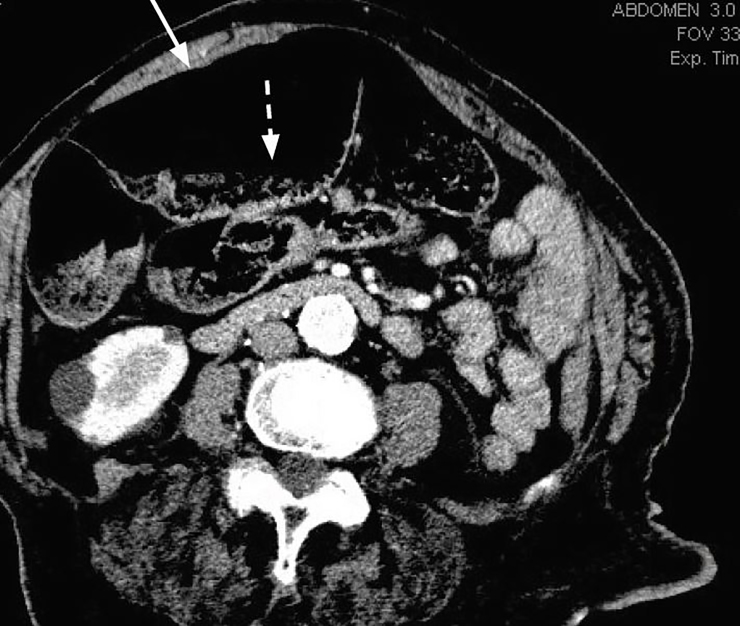
Computed tomography axial image demonstrating gaseous distension of the cecum (solid white arrow) with visualized air-fluid levels within the bowel (dashed white arrow).

A computed tomography (CT) of her abdomen and pelvis led to the diagnosis of a cecal bascule, and general surgery was consulted (Images 2 and 3). The patient received a right hemicolectomy and ileostomy that evening and was admitted to the surgical intensive care unit following the procedure.

## DISCUSSION

Cecal volvulus has been described as a cause of intestinal obstruction since first noted in 1837 by Rokitansky. 4 Failure of fixation of the cecum in the right lower quadrant during embryogenesis can predispose an individual to formation of a volvulus.^1^ The cecal bascule, which is far less common, was defined by Weinstein in 1938 as a subtype of cecal volvulus accounting for less than 10% of all cases.^5^

Cecal bascule, caused by the cecum folding anterior to the ascending colon, leads to intestinal obstruction. 6 This anterior folding causes inflammatory adhesions to form between the original anterior wall and the ascending colon.^4^ This in turn leads to a flap valve occlusion that prevents cecal emptying. 4 Distension of the cecum then occurs proximal to this outlet obstruction. If there is a competent ileocecal valve, there is no retrograde decompression of the cecum into the small bowel, and this leads to subsequent gaseous and fluid distension. 4

**CPC-EM Capsule**

What do we already know about this clinical entity?

Cecal bascule is a rare type of bowel obstruction that requires early surgical intervention to prevent life-threatening complications.

What makes this presentation of disease reportable?

Our case demonstrates how a cecal bascule could potentially present in the ED and the necessary work-up required to make this important diagnosis.

What is the major learning point?

With its varied presentations and intermittent symptoms, a cecal bascule is a difficult diagnosis that requires a high level of clinical suspicion.

How might this improve emergency medicine practice?

By understanding the unique clinical presentation of a cecal bascule, emergency physicians will be more apt to make the diagnosis and intervene accordingly.

Cecal volvulus most commonly occurs in young women during the postpartum period when the cecum is displaced upward by the uterus, or in elderly hospitalized patients secondary to reduced intestinal mobility. 6 Chronic constipation, high fiber consumption, and prolonged immobilization are thought to be risk factors for this condition.^7^,^8^ The presence of adhesions, most commonly from previous abdominal surgery, can also contribute to the formation of points of fixation that can act as rotation axes. 6

Even though there is no torsion of the mesentery in a cecal bascule, cecal distension can still cause venous and capillary constriction leading to ischemic changes that can progress to gangrene.^9^ Hence, early surgical decompression must be a priority in the management of these patients. Though of critical importance, there is no set standard on how decompression is achieved. It can take numerous forms including colectomy, cecopexy, cecopexy supplemented by cecostomy, or cecectomy.^10^

Cecal bascule is a difficult condition to diagnose; thus, a high index of clinical suspicion must be maintained. Clinical manifestations include nausea, vomiting and continuous abdominal pain with exacerbation of pain during peristaltic movement. 6 Unfortunately, there is no discussion in the literature concerning how long these exacerbations last or whether patients are ever completely symptom-free. On exam, these patients will have a distended tympanic abdomen.^9^ As vascular compromise progresses, these patients may begin to demonstrate signs of peritoneal irritation. 11

Laboratory results are neither sensitive nor specific, but practitioners typically find a leukocytosis and potentially hyperkalemia, azotemia or anemia in more severe cases.^10^ In our patient the lack of these findings likely portended to her having presented early in the disease course, and her hypokalemia and alkalosis resulted from her being relatively volume depleted -- not from vomiting as is the usual case, but from being unable to use her PEG-tube as it was causing her discomfort.

Abdominal radiography may illustrate air-fluid levels with dilation of small-bowel loops and a predominate distension of the cecum that is displaced anteromedial to the transverse colon.^12^ Computed tomography (CT) is the preferred imaging technique to confirm a cecal bascule.^9^ Radiological signs on CT may include a coffee bean or comma sign seen on axial imaging.^6^ CT also enables visualization of the obstruction site, and the presence of wall thickening, mesenteric fat trabeculation, intestinal pneumatosis, or free fluid within the pelvis that can indicate imminent bowel ischemia.^6^

Diagnostic colonoscopy is not recommended as it may increase the risk of perforation.^6^ Right hemicolectomy with ileotransverse anastomosis is the preferred surgical technique as it carries the lowest risk of recurrence and a lower rate of morbidity and mortality when compared to detorsion alone, detorsion with cecopexy, and cecostomy.^6^,^13^ If anastomosis is not possible, an ileostomy should be performed.^6^

The purpose of this case report is to remind emergency physicians to expand their differential diagnosis to include the less-common causes of abdominal pain, such as cecal bascule, especially in postpartum women and the elderly. Cecal bascule is a rare cause of intestinal obstruction and is a uniquely challenging diagnosis due to the intermittent nature of its presentation. The periodic cephalad folding of the cecum allows variation in clinical signs and imaging findings depending on the positioning of the cecum at that specific point in time. Therefore, this diagnosis must remain on the differential of any patient reporting signs of intestinal obstruction even if not clinically evident at that moment.

## CONCLUSION

Cecal bascule is a rare cause of bowel obstruction where there is anterior displacement of the cecum over the ascending colon. Making this diagnosis is especially difficult due to the relatively non-specific constellation of symptoms that may or may not include distension, abdominal pain, tenderness to palpation, nausea, vomiting, hyperperistalsis and absent bowel sounds. A high level of clinical suspicion and early surgical intervention are essential in preventing the devastating complications of bowel ischemia and gangrene.

Documented patient informed consent and/or Institutional Review Board approval has been obtained and filed for publication of this case report.
